# The Outcome of Health Anxiety in Primary Care. A Two-Year Follow-up Study on Health Care Costs and Self-Rated Health

**DOI:** 10.1371/journal.pone.0009873

**Published:** 2010-03-24

**Authors:** Per Fink, Eva Ørnbøl, Kaj Sparle Christensen

**Affiliations:** 1 The Research Clinic for Functional Disorders and Psychosomatics, Aarhus University Hospital, Aarhus, Denmark; 2 The Research Unit for General Practice, Aarhus University, Aarhus, Denmark; James Cook University, Australia

## Abstract

**Background:**

Hypochondriasis is prevalent in primary care, but the diagnosis is hampered by its stigmatizing label and lack of valid diagnostic criteria. Recently, new empirically established criteria for Health anxiety were introduced. Little is known about Health anxiety's impact on longitudinal outcome, and this study aimed to examine impact on self-rated health and health care costs.

**Methodology/Principal Findings:**

1785 consecutive primary care patients aged 18–65 consulting their family physicians (FPs) for a new illness were followed-up for two years. A stratified subsample of 701 patients was assessed by the Schedules for Clinical Assessment in Neuropsychiatry interview. Patients with mild (N = 21) and severe Health anxiety (N = 81) and Hypochondriasis according to the DSM-IV (N = 59) were compared with a comparison group of patients who had a well-defined medical condition according to their FPs and a low score on the screening questionnaire (N = 968). Self-rated health was measured by questionnaire at index and at three, 12, and 24 months, and health care use was extracted from patient registers. Compared with the 968 patients with well-defined medical conditions, the 81 severe Health anxiety patients and the 59 DSM-IV Hypochondriasis patients continued during follow-up to manifest significantly more Health anxiety (Whiteley-7 scale). They also continued to have significantly worse self-rated functioning related to physical and mental health (component scores of the SF-36). The severe Health anxiety patients used about 41–78% more health care per year in total, both during the 3 years preceding inclusion and during follow-up, whereas the DSM-IV Hypochondriasis patients did not have statistically significantly higher total use. A poor outcome of Health anxiety was not explained by comorbid depression, anxiety disorder or well-defined medical condition. Patients with mild Health anxiety did not have a worse outcome on physical health and incurred significantly less health care costs than the group of patients with a well-defined medical condition.

**Conclusions/Significance:**

Severe Health anxiety was found to be a disturbing and persistent condition. It is costly for the health care system and must be taken seriously, i.e. diagnosed and treated. This study supports the validity of recently introduced new criteria for Health anxiety.

## Introduction

Hypochondriasis is a rarely used diagnosis in clinical practice despite studies having reported prevalence between 0.8–9.5% in primary care [1]–[3]. This may be because the disorder is not taken seriously but rather is viewed as an imaginary illness or a phenomenon secondary to another psychiatric disorder [4], [5]. Hypochondriasis is also considered a stigmatizing label, and the designation ‘Health anxiety’ has been suggested as replacement and is used in this paper.

There is some evidence that numerous somatic symptoms or illness worry may be associated with impairment and high health care utilization [3], [6]–[14]. However, most studies have used self-reported questionnaires and/or layman interviews on the basis of which clinical diagnoses cannot be established. Also, with a few exceptions, the studies are retrospective in design, and comorbidity with other mental or physical disorders has not been taken into account. We are not aware of any longitudinal studies on health care costs or self-rated health that followed up patients with a Hypochondriasis diagnosis according to Diagnostic and Statistical Manual (DSM-IV) or International Classification of Diseases (ICD-10). Relatively little is thus known about Hypochondriasis' impact on self-rated functioning related to mental and physical health and longitudinal outcome [15]–[17].

The lack of valid, reliable, and generally accepted diagnostic criteria has been a major obstacle in clinical practice and Hypochondriasis studies [1], [4], [5], [18]–[20]. Recently, we introduced a radically revised definition of Hypochondriasis, which we call Health anxiety to decrease the pejorative connotation. This new diagnosis is empirically established and has shown to be more valid than the ICD-10 and DSM-IV definitions, both from a clinical and a nosological viewpoint [2]. The new diagnostic criteria include the symptom ‘obsessive rumination about illness’ plus at least one of the symptoms ‘worry or preoccupation with fears of harboring an illness or with bodily functions’, ‘suggestibility or autosuggestibility’, ‘an unrealistic fear of being infected or contaminated’, ‘an excessive fascination with medical information’, or ‘fear of taking prescribed medication’ (Table S1). If the patient has a comorbid medical condition, the symptoms must not be accounted for by this. The patients are divided into ‘mild’ and ‘severe’ groups, the latter being characterized by clinically significant distress or functional impairment [2]. We conducted a two-year follow-up study of patients with Health anxiety defined either as fulfilling criteria for DSM-IV Hypochondriasis and/or the recently introduced empirically established Health anxiety diagnosis [2] (Table S1). We used the patient sample in which the new (diagnostic) criteria were developed [2]. We examined the diagnosis' clinical importance by assessing its ability to predict outcome in terms of Health anxiety persistence (Whiteley-7 scale), the disorder's impact on self-rated functioning related to mental and physical health (SF-36 mental and physical component scores (MCS and PCS)), and health care costs, and we controlled outcome for the effect of comorbid depression or anxiety disorder. The outcomes of patients with Health anxiety were compared to outcomes of patients with a well-defined medical condition.

## Methods

### Ethics statement

This study was approved by the Science Ethics Committee of the Central Denmark Region.

We received written informed consent from all the participating patients.

### Patients and setting

**Figure 1:**
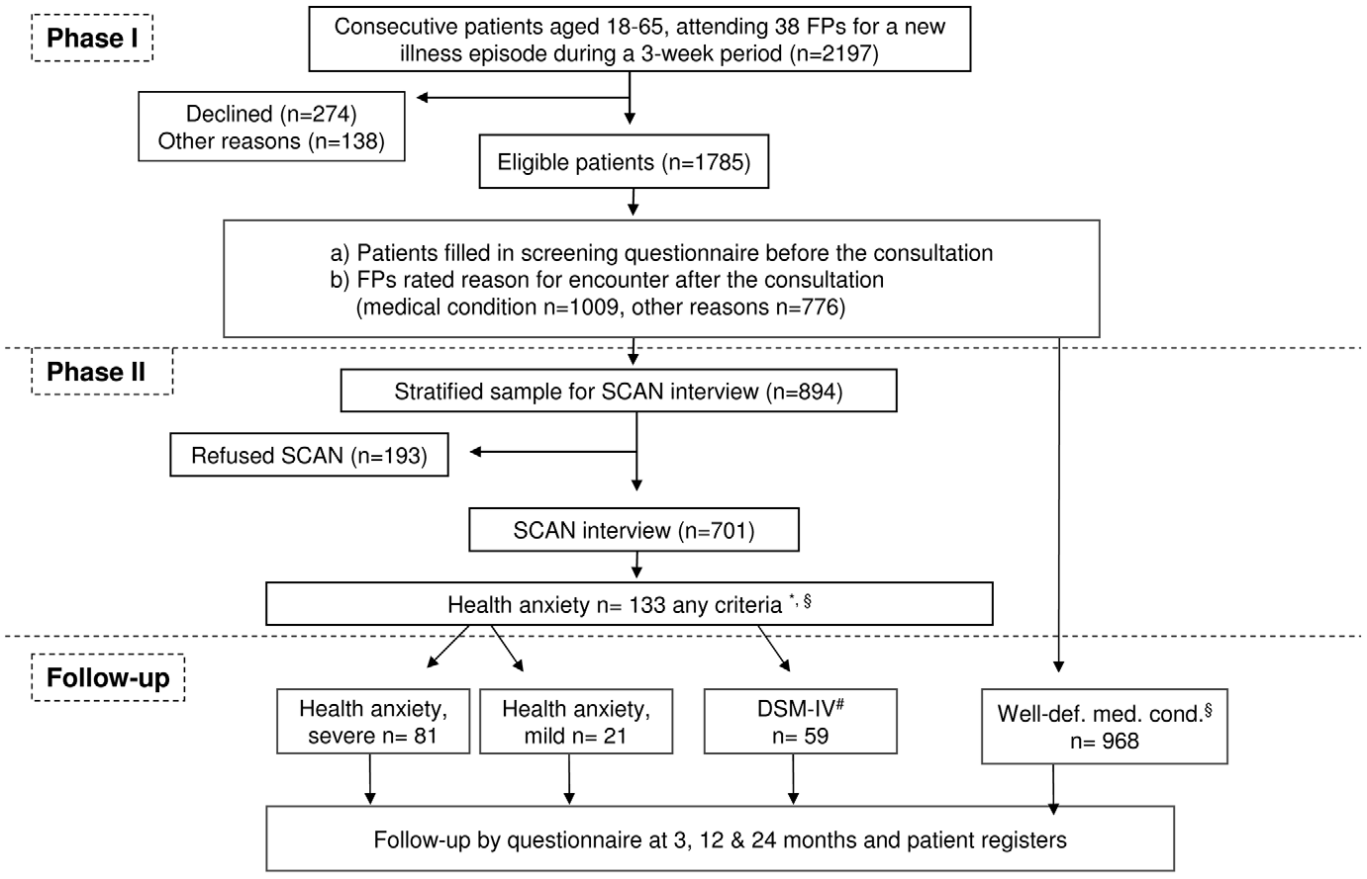
Flowchart. £ 28 patients both had Health anxiety (severe or mild) and DSM-IV Hypochondriasis. § 41 of the Health anxiety had a well-defined medical condition as reason for encounter. To avoid overlap, they were excluded from the medical condition group. * Responses to questionnaires (Whiteley-7 and SF-36). # DSM-IV Hypochondriasis.

This study was based on a randomized controlled trial (RCT) on the effect of training family physicians (FPs) in treating patients presenting with medically unexplained symptoms [21]. The study included 1785 (aged 18–65 years) consecutive patients of Scandinavian origin consulting their FP during a three-week period for a new illness problem. 2197 patients, of whom 274 declined the invitation to participate, met the predefined inclusion and exclusion criteria (exclusion criteria: too ill or demented to read and fill in questionnaires, exclusively consulting for health check, or not listed with the practice), and 138 did not participate for other reasons (Figure 1). The mean age of those declining participation was 42.2 compared with 38.8 for included participants (t-test, t (2046) = −4.0 p<0.001). There was no significant gender difference between the two groups. The sociodemographic characteristics of the sample have been reported elsewhere [2]. All participants were covered by the National Health Service, which includes 98% of the Danish population, all individuals being registered with a specific FP.

### Study design and procedures

We used a two-phase design. First, all patients filled in a screening questionnaire in the waiting room (Figure 1). This questionnaire included among others the eight-item version of the Symptom Check List (SCL-8d) [22], [23] assessing anxiety and depression, the seven-item Whiteley scale [24] measuring worrying and conviction of illness, the somatization subscale of the SCL-90 (SCL-SOM) checking for 12 common physical symptoms [25], and the Cutting down, Annoyance by Criticism, Guilty feeling, Eye openers questionnaire (CAGE) which consists of four questions screening for alcohol abuse [26]. The patients also filled in the Medical Outcome Study's Short Form (SF-36) [27].

Patients with a total score of two or more on the SCL-8d, or the Whiteley-7, or the CAGE, or 4 or more on the SCL-SOM were selected for the second phase; a diagnostic psychiatric interview. Furthermore, a random sample of 1/9 of the remaining patients was selected for interview to produce a stratified subsample consisting of all high-scorers and 1/9 of the low-scorers. 894 patients were selected for interview, but 193 (21.6%) declined the invitation. A comparison of the patients declining interview with the 701 interviewees showed that the former group was composed of more men, was younger, and had more missing answers on sociodemographic variables. Fewer from the group declining interview were employed, were living with a partner, or had an education beyond basic school compared with the interviewees, and the former group also had lower scores on the somatic symptom checklist (SCL-SOM) than interviewees (for details see [2], [28]).

### The psychiatric research interview

The psychiatric interviews were conducted as soon as possible after the index contact, either in the FPs' clinics, in the research clinic, or in the patients' own homes.

Research interviewers used the Schedules for Clinical Assessment in Neuropsychiatry (SCAN) version 2.1 [29], [30], which is a standardized, semi-structured, comprehensive interview endorsed by the World Health Organization (WHO). It covers all types of mental disorders. The interview includes an extensive section on physical health, which is not primarily designed to assess physical conditions but somatoform and similar disorders. The interview lasts from 30 minutes in patients with no health problems to several hours in patients with severe problems. The interviewers were free to explore external sources of information when they needed clarification of the interview, for instance by reviewing medical records. This is helpful if the interview fails to disclose if a physical complaint is medically unexplained or attributable to a well-defined medical condition, and it is standard procedure when using the SCAN. To avoid introducing any bias, the interviewers were not allowed to request information from the patients' own FPs. External information was requested for only few patients.

The section on physical health in the SCAN interview was extended with additional items on Health anxiety symptoms (for details see [2]). The SCAN interview requires psychiatric interviewing skills, and in this study, six psychiatric doctors certified at the WHO centre in Copenhagen conducted them. An analysis of eight SCAN interviews which were rated by all six interviewers showed a high inter-rater agreement on the presence of/lack of a somatoform diagnosis (kappa = 0.82) [28]. Furthermore, five of the six interviewers agreed on a severe Health anxiety and a DSM-IV Hypochondriasis diagnosis in one patient (one interviewer did not rate this patient), whereas none of the other patients were rated as having Health anxiety according to any criteria. As we did not find disagreement among the interviewers, we were unable to calculate a Kappa value concerning inter-rater agreement of Health anxiety. The interviewers were blinded to the patients' responses on the screening questionnaires.

### FP assessment

After the index consultation, the FPs rated whether the consultation was due to ‘well-defined medical condition’ (n = 1009), ‘probably well-defined medical condition’ (n = 395), ‘medically unexplained somatic symptoms’ (n = 229), ‘well-defined mental disorder with somatic symptoms’ (n = 95), or ‘no physical problem’ (n = 39). In 18 patients, the FPs had not rated the reason for encounter.

### Follow-up

All patients received a questionnaire by mail at three, 12, and 24 months after the index consultation. The questionnaire included among other items the Whiteley-7 and the SF-36. Non-completers received a reminder after two weeks. For both follow-up and index it was required that patients answered at least 50% of the items in each scale or index to obtain a score. Otherwise, the score was set to missing.

### Health care costs

The Danish health care system is almost entirely tax financed and, with a few exceptions, all medical care is free of charge. We obtained costs of primary and specialized care and psychiatric and general hospital inpatient and outpatient care from the National Health Service register in the County of Aarhus from a period of three years preceding inclusion through two years after inclusion. Costs of prescribed medicine were obtained from the National Health Service register in the County of Aarhus for a period of six months preceding inclusion through two years after inclusion in the study. Limitations in the Danish law on medicine use registration prevented us from obtaining retrospective data on prescribed medicine for a longer period. Costs of lab tests and x-rays prescribed in primary care were not included.

All costs for inpatient and daytime admissions and outpatient and emergency ward contacts were extracted from the Danish National Patient Register and The Danish Psychiatric Central Register [31]. Non-psychiatric costs were calculated as Diagnosis Related Group case-mix (DRG) prices by the DRG pricing office of the Danish National Board of Health in 2004 fixed prices. Psychiatric hospital care costs were calculated from average 2004 fixed prices for hospital bed days and outpatient contacts with aid from the economic administration of Psychiatric Hospital in Aarhus as the hospital is not using DRG prices. All costs were adjusted for time at risk, and the object for analysis was cost per year. Index consultation was included in the two years after index.

### Comparison with general population sample

We had access to two Danish representative general population surveys, one on validation of the SF-36 [32], the other on validation of the SCL-90 [33]. From each of these population surveys, we constructed a sample matched for age and gender with the primary care sample.

### Data analysis

The SCAN interviews were used for computerized DSM-IV psychiatric diagnoses, including Hypochondriasis according to DSM-IV criteria. The section on physical health in the SCAN interview was processed separately in order to make the Health anxiety diagnosis.

We compared patients with DSM-IV Hypochondriasis (n = 59) and mild (n = 21) and severe (n = 81) Health anxiety (Table S1) with patients having a well-defined medical condition according to the FPs. 296 of the 1009 patients who according to the FPs consulted due to a well-defined medical condition were SCAN interviewed, and the SCAN detected that 41 of those patients also had Health anxiety or DSM-IV Hypochondriasis. To avoid inclusion of patients in both the medical condition group and one of the Health anxiety groups, those 41 patients were only included in one the health anxiety groups and excluded from the medical condition group, and therefore this group includes 968 patients in total (Figure 1). 28 patients fulfilled the diagnostic criteria for both DSM-IV Hypochondriasis and Health anxiety (Table 1) [2]. Thus, 133 patients had Health anxiety or DSM-IV Hypochondriasis. The Whiteley-7 scale is used in different versions in the medical literature, and to make comparison between different versions easier, we transformed the scores of the Whiteley-7 into a scale ranging from 0–100 by the expression (a patient's actual score–lowest possible score (i.e. 0))/(Highest possible score (i.e. 28)–lowest possible score (i.e. 0)) × 100. The same method was used for the SF-36 questionnaire [34]. As the study was part of a randomized controlled study on the effect of training FPs in treating patients presenting with medically unexplained symptoms [35], [36], we controlled for intervention in the analyses. Group comparison was computed by χ^2^ tests for categorical data, and Mann-Whitney U or Kruskal-Wallis test was used for non-normally distributed continuous data.

**Table 1:** Characteristics of the sample.

	Health anxiety				DSM-IV Hypochondriasis		Well-defined	
	Severe		Mild				medical cond.*	
	(a)		(b)		(c)		(d)	
Number of patients	81		21		59		968	
Females%†	71.6		61.9		79.6		58.9	
Age (mean, SD)‡	39.7	13.1	39.1	13.1	38.4	11.3	37.8	13.1
Comorbidity	n	%	n	%	n	%	n	%
Major depressive episode	14	17.3	1	4.8	9	15.3	9	3.5
Anxiety disorder	29	35.8	1	4.8	25	42.2	20	7.8
Medical condition according to FP								
Definitely	20	24.7	7	33.3	19	32.2	-	
Probably	22	27.2	6	28.6	13	22.0	-	
DSM-IV Hypochondriasis	25	30.9	3	14.3	-	-	-	-
Health anxiety								
Mild	-	-	-	-	3	5.1	-	-
Severe	-	-	-	-	25	42.4	-	-

*The FPs rated definitely well-defined medical conditon as reason for encounter at index and the patients had no Health anxiety at diagnostic interview.

†χ2 test: Health anxiety (a,b) vs medical condition (d) χ2 = 5.1, p = 0.079, df = 2, DSM-IV hypochondriasis vs medical condition c2 = 10.0, p = 0.002, df = 1.

‡: Health anxiety (a,b) vs medical condition (d), Kruskal-Wallis test: χ2 = 2.0, p = 0.3683, df = 2, DSM-IV Hypochondriasis vs medical condition, Mann-Whitney test, Z = −0.75, p = 0.456.

We wanted to compare patients with Health anxiety or DSM-IV Hypochondriasis with patients having a well-defined medical condition according to their FPs, thereby estimating two models for each outcome. Inspecting the patients' individual graphs for Whiteley-7, PCS, and MCS over time, we worked out that an appropriate choice of model was a mixed effect model with random intercept in order to model the mean differences in scores [37]. The general shape of the model is a group-specific level at index and a group-specific level, i.e. one level, at the remaining time points. This was modelled through two variables with a possible interaction. We included linear adjustment for gender, age, comorbid major depressive or anxiety disorder, medical condition, and intervention. A Wald test for interaction between the Health anxiety diagnosis and either comorbid major depressive or anxiety disorder was performed. The model was checked by diagnostic plots of the residuals.

In the lack of general population norms for response on the Whiteley-7 scale, we arbitrarily set the cutpoint for a normal versus “ill” response equal to the 90 percent percentile at 24-month follow-up for patients with a well-defined medical condition.

In order to account for skewed health care costs with an excess of zeros, we have estimated sample means of health care costs with bias-corrected and accelerated (BC_a_) 95% confidence intervals. Test of equality of health care cost means for patients with a medical condition according to their FP and patients with Health anxiety were done by computing the bootstrap test statistic achieved significance level (ASL) [38].

We processed the data in STATA 9 [39].

## Results

The diagnostic group characteristics and comorbidity between diagnoses are shown in Table 1. As 28 patients fulfilled diagnostic criteria for both Health anxiety and DSM-IV Hypochondriasis, the combined final sample size was 133, i.e. patients who either had Health anxiety or DSM-IV Hypochondriasis. Between 24.7 and 33.3% of the patients with Health anxiety according to the diagnostic interview definitely had a well-defined medical condition as reason for encounter according to their FPs. The Health anxiety group comprised more females than the patient group with a well-defined medical condition. No age difference was found between groups.

Health anxiety measured on the Whiteley-7 scale is displayed in Table 2. The highest number lost to follow-up was in the DSM-IV Hypochondriasis group in which only 66% answered the 24-month follow-up questionnaire. At least 78% of the patients with severe Health anxiety completed the questionnaire, in the mild Health anxiety group 87% or more did, and in the medical condition group 72% or more answered the follow-up questionnaire. There were no statistically significant differences in Whiteley-7 scores at index consultation between patients completing the questionnaires and patients declining to complete at any follow-up times (Mann-Whitney U test, p>0.086 or higher) (details available from the authors on request).

**Table 2:** Outcome of health anxiety on Whiteley-7 scale (scores 0–100) during two-year follow-up.

	Health anxiety								DSM-IV Hypochondriasis				Medical condition					
	Severe (N = 81) (a)				Mild (N = 21) (b)				DSM-IV (N = 59) (c)				(N = 968) (d)			Mann-Whitney test		
	n	median	Q1–Q3	%*	n	median	Q1–Q3	%*	n	median	Q1–Q3	%*	n	median	Q1–Q3	a vs d	b vs d	c vs d
Index	81	28.6	14.3–42.9	59.3	20	25.0	19.6–35.7	60.0	59	28.6	17.9-42-9	55.9	963	3.6	0–14.3	p<0.0001	p<0.0001	p<0.0001
3 mths	65	21.4	14.3–35.7	47.7	17	10.7	3.5–17.9	17.6	48	21.4	14.3–37.5	47.9	697	3.6	0–10.7	p<0.0001	p = 0.0213	p<0.0001
12 mths	63	21.4	7.1–32.1	42.9	18	14.3	3.6–17.9	16.7	42	21.4	7.1–32.1	45.2	690	0.0	0–10.7	p<0.0001	p = 0.0138	p<0.0001
24 mths	64	21.4	5.4–39.3	45.3	20	12.5	1.9–25	30.0	39	25.0	7.1–35.7	51.3	699	0.0	0–10.7	p<0.0001	p = 0.0078	p<0.0001

Q1 = 25th percentile, Q3 = 75th percentile.

*Percentage of patients who score above 21.5 (ie. the 90% percentil of patient with a wel-defined medical condition at 24 months).

**Figure 2:**
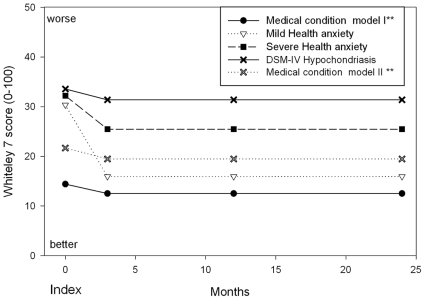
Health anxiety during follow-up according to mixed effect model with a random intercept. * Corrected for age, gender, comorbid anxiety disorder, or major depression, medical condition and intervention. ** Statistics displayed in Table 3. Health anxiety and DSM-IV Hypochondriasis were tested in two separate statistical models, and the expected values for the medical condition groups were not identical despite including the same individuals. Model 1 is for Health anxiety vs medical condition including 1066 patients with 3393 scores, model 2 is DSM-IV Hypochondriasis vs medical condition including 1024 patients with 3237 scores.

The results from the two mixed effect models for Whiteley-7 are shown in Table 3 and Figure 2. The scores of both the severe and mild Health anxiety and the DSM-IV Hypochondriasis patients were statistically significantly higher than the scores for the patients with a well-defined medical condition according to the FPs at index consultation. Health anxiety scores dropped significantly from index consultation to later follow-up in all diagnostic groups. At later follow-up times, the Whiteley-7 scores remained at a statistically significantly higher level in the severe Health anxiety and the DSM-IV Hypochondriasis patients compared with patients with a well-defined medical condition, whereas there were no statistically significant differences between the mild Health anxiety patients and those with a well-defined medical condition (Table 3 and Figure 2). We tested for interaction with comorbid major depressive and anxiety disorder, but none were statistically significant.

**Table 3:** Results from mixed effect models with random intercept.

	Change in mean score from index index to later time points	95% CI	P-value	Difference in mean score at index *	95% CI	P-value	Difference in mean score at later time points*	95% CI	P-value
**Whiteley-7**									
Well-defined medical cond. 1	−1.90	(−2.60;−1.21)	<0.001						
Health anxiety, mild	−14.42	(−20.76;−8.09)	<0.001	15.95	(7.78;24.12)	<0.001	3.43	(−2.84;9.70)	0.283
Health anxiety, severe	−6.73	(−10.99;−2.47)	0.002	17.79	(11.23;24.35)	<0.001	12.96	(6.85;19.07)	<0.001
Well-defined medical cond. 2	−2.19	(−2.91;−1.46)	<0.001						
DSM-IV Health anxiety	−2.19	(−2.91;−1.46)	<0.001	11.89	(6.10;17.69)	<0.001	11.89	(6.10;17.69)	<0.001
**PCS**									
Well-defined medical cond. 1	2.35	(1.88;2.81)	<0.001						
Health anxiety, mild	2.35	(1.88;2.81)	<0.001	1.37	(−2.40;5.14)	0.477	1.37	(−2.40;5.14)	0.477
Health anxiety, severe	2.35	(1.88;2.81)	<0.001	−4.56	(−8.28;−0.85)	0.016	−4.56	(−8.28;−0.85)	0.016
Well-defined medical cond. 2	2.41	(1.94;2.87)	<0.001						
DSM-IV Hypochondriasis	2.41	(1.94;2.87)	<0.001	−4.1	(−7.51;−0.69)	0.018	−4.1	(−7.51;−0.69)	0.018
**MCS**									
Well-defined medical cond. 1	−0.71	(−1.27;−0.17)	0.011						
Health anxiety, mild	4.07	(−1.08;9.22)	0.121	5.26	(−0.86;11.39)	0.092	0.48	(−3.67;4.63)	0.822
Health anxiety, severe	1.66	(−0.78;4.09)	0.181	6.61	(2.87;10.35)	0.001	4.23	(0.70;7.78)	0.019
Well-defined medical cond. 2	−0.71	(−1.27;−0.17)	0.011						
DSM-IV Hypochondriasis	3.31	(0.49;6.13)	0.021	7.56	(3.42;11.71)	<0.001	3.54	(−0.49;7.57)	0.085

*The differences are mean scores for the Health anxiety groups minus the mean score for the well-defined medical condition group.

**All estimates concerning one outcome are based on two mixed effect models with random intercept and linear adjustment for gender, age at baseline, comorbid major depressive or anxiety disorder or medical condition, and intervention.

Results stated as change in mean score within diagnosis groups from index to later time points and differences in mean scores between diagnosis groups both at index and at later time points.

Table 2 also displays the percentage of patients scoring above 21.5 on the Whiteley-7 scale. It is seen that 45.3% of the patients with severe Health anxiety would still be ill at 24 months using this cutpoint for an “ill” response. However, it has to be noticed that only 59.3% of the Health anxiety patients scored above this cutpoint at the index consultation and thus the fraction of patients above the cutpoint score seems quite stable in that very few Health anxiety patients seem to get well.

The PCS and MSC scores appear from Table 4. Patients with mild Health anxiety and patients with a well-defined medical condition had scores similar to the age and gender matched general population sample. Some patients were lost to follow-up; the highest number in the well-defined medical condition group in which only 60% answered the 24-month follow-up questionnaire, whereas 66% or more of the DSM-IV Hypochondriasis patients, 65% or more of the severe Health anxiety, and at least 76% of the mild Health anxiety patients completed the follow-up questionnaire. There were no statistically significant (p>0.36, Mann-Whitney U test) differences in PCS or MCS scores at index between patients completing the questionnaires and non-completers, nor at any of the follow-up periods.

**Table 4:** SF-36 physical and mental component (PCS/MCS) summary scores during two-year follow-up and scores in age and gender matched normal population.

		Health anxiety						Hypochondriasis			Medical condition					
		Severe (N = 81) (a)			Mild (N = 21) (b)			DSM-IV (N = 59) (c)			(N = 968) (d)			Mann-Whitney test		
		n	mean	SD	n	mean	SD	n	mean	SD	n	mean	SD	a vs d	b vs d	c vs d
**PCS**																
	Index	72	42.9	12.8	19	50.3	8.3	54	42.9	10.0	788	49.3	9.1	p<0.0001	p = 0.7110	p<0.0001
	3 months	63	44.1	11.5	17	51.2	5.6	47	44.0	10.6	687	51.3	8.8	p<0.0001	p = 0.2416	p<0.0001
	12 months	61	45.6	11.2	16	51.6	6.8	41	45.3	11.0	675	51.6	8.4	p<0.0001	p = 0.5720	p<0.0001
	24 months	53	44.9	11.5	17	51.7	5.2	39	45.5	9.6	579	51.5	8.1	p<0.0001	p = 0.48110	p = 0.0001
**MCS**																
	Index	72	41.3	13.2	19	45.0	13.6	54	42.4	12.2	788	53.9	8.5	p<0.0001	p = 0.0006	p<0.0001
	3 months	63	45.0	11.4	17	50.2	9.8	47	44.6	13.9	687	53.8	8.9	p<0.0001	p = 0.0552	p<0.0001
	12 months	61	42.4	12.2	16	48.6	9.8	41	45.3	11.9	675	53.0	9.1	p<0.0001	p = 0.0228	p<0.0001
	24 months	53	42.5	13.2	17	48.5	11.0	39	45.1	12.2	579	53.0	9.3	p<0.0001	p = 0.0470	p<0.0001
		Age and gender														
		matched normal pop.														
		n	mean	SD												
**PCS**		2111	52.2	8.0												
**MCS**		2111	53.5	8.4												

The results from the two mixed effect models for PCS mean scores are provided in Table 3. All diagnostic groups had statistically significant improvements in PCS scores from index to later follow-up times. The differences in PCS scores between the severe Health anxiety patients, the DSM-IV Hypochondriasis patients, and the well-defined medical condition patients were statistically significant (Table 3). We found no statistically significant interactions with comorbid major depressive and anxiety disorders and the Health anxiety and DSM-IV diagnoses.

**Figure 3:**
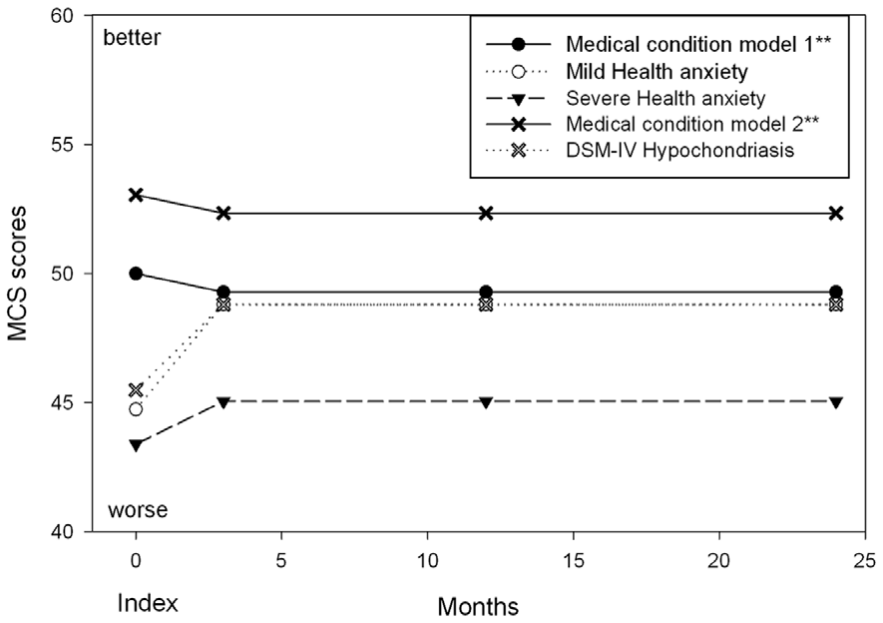
Mental component summary score during follow-up according to mixed effect model with a random intercept. * Corrected for age, gender, comorbid anxiety disorder, or major depression, medical condition and intervention. ** Statistics displayed in Table 3. Health anxiety and DSM-IV Hypochondriasis were tested in two separate statistical models, and the expected values for the medical condition groups were not identical despite including the same individuals. Model 1 is for Health anxiety vs medical condition including 984 patients with 3042 scores, model 2 is DSM-IV Hypochondriasis vs medical condition including 944 patients with 2910 scores.

Functioning related to mental health did not improve significantly over time in the mild and severe Health anxiety patients, whereas the DSM-IV Hypochondriasis patients improved statistically significantly after the index consultation (Table 3 and Figure 3). Patients with severe Health anxiety and DSM-IV Hypochondriasis had significantly lower MCS scores at three-month follow-up compared with the well-defined medical condition patients. Test for interaction of comorbid major depressive and anxiety disorders revealed statistical significance between DSM-IV Hypochondriasis and major depressive episode (Wald test, χ(1) = 4.79, p = 0.029). A comorbid major depressive episode had a more severe impact on patients with a well-defined medical condition than on patients with DSM-IV Hypochondriasis.

The diagnostic plots of the residuals were examined for the six models presented in Figure 2, 3, and Table 3, and no critical departure from the model stated normal distributions was seen.

### Health care utilization

The overall health care costs and use of primary care for the period of three years preceding the index consultation through two years after index was quite constant for all patient groups (data not shown). The mean health care costs per year in Euro appear from Table 5. Overall, the severe Health anxiety patients used about 41–78% more health care services, excluding laboratory tests and x-rays prescribed in primary care, per year than patients with a well-defined medical condition, both before and after the encounter in primary care. The former group's use was statistically significantly different from that of the latter group for all types of health care use displayed in Table 5, except for general hospital and psychiatric care after the index consultation. The DSM-IV Hypochondriasis patients had a significantly higher use of primary care compared with the well-defined medical condition patients, whereas there were no statistically significant differences as to the other types of use displayed in Table 5. The medicine reimbursement stemming from psychoactive drugs accounted for 22% in patients with a well-defined medical condition and 38% in patients with severe Health anxiety or DSM-IV Hypochondriasis (results not shown). Contrary to patients with severe Health anxiety, patients with mild Health anxiety use significantly less health care than patients with a well-defined medical condition.

**Table 5:** Use of health care per year before and after index consultation in Euro*.

	Health anxiety												Hypochondriasis						Well-def. med. cond.						Test for equality of means		
Health service costs	Severe (N = 81)						Mild (N = 21)						DSM-IV (N = 59)						(N = 968)						a vs d	b vs d	c vs d
	(a)						(b)						(c)						(d)								
	Mean	BCa 95% CI					Mean		BCa 95% CI				Mean		BCa 95% CI				Mean		BCa 95% CI				ASL	ASL	ASL
**Overall**																											
before	1766	(	1321	:	2572	)	512	(	317	:	761	)	1200	(	937	:	1545	)	989	(	896	:	1161	)	<0.001	0.002	0.094
after	1533	(	1232	:	1974	)	672	(	412	:	1061	)	1099	(	840	:	1488	)	1086	(	923	:	1386	)	0.021	0.046	0.46
**Primary care**																											
before	262	(	216	:	363	)	152	(	109	:	218	)	265	(	216	:	347	)	129	(	119	:	145	)	<0.001	0.167	<0.001
after	310	(	248	:	433	)	162	(	119		223	)	275	(	230	:	355	)	167	(	154	:	186	)	<0.001	0.426	<0.001
**Medicine reimbursement** #																											
before	301	(	187	:	513	)	79	(	42	:	147	)	239	(	147	:	518	)	156	(	128	:	190	)	0.007	0.019	0.059
after	228	(	139	:	415	)	45	(	20	:	108	)	156	(	96	:	263	)	105	(	85	:	131	)	<0.001	0.2	0.084
**General hospital care**																											
before	927	(	674	:	1350	)	244	(	130	:	499	)	641	(	459	:	909	)	660	(	576	:	779	)	0.036	0.001	0.439
after	907	(	662	:	1229	)	465	(	214	:	882	)	576	(	368	:	836	)	770	(	615	:	1005	)	0.241	0.084	0.103
**Psychiatric care**																											
before	275	(	48	:	1143	)	37	(	0	:	186	)	54	(	14	:	151	)	44	(	14	:	155	)	0.076	0.479	0.423
after	88	(	40	:	189	)	0	(	0	:	0	)	92	(	31	:	215	)	45	(	6	:	165	)	0.27	0.026	0.247

*The ‘after’ period includes the month of the index consultation, 1 Euro = 7.59 dkr.

# Only 1 year before index.

BCa  =  bias-corrected and accelerated.

ASL  =  achieved significance level.

## Discussion

This study suggests that Health anxiety in its severe form has significant long-term impact on the patients' self-rated functioning related to mental and physical health and on health care costs, and the patients persistently report high levels of Health anxiety measured on Whiteley-7. Health anxiety in its mild form, however, seems not to have any significant negative impact on physical health and health care costs.

We used two definitions of “caseness”: the DSM-IV Hypochondriasis criteria and the newly introduced empirically established criteria for Health anxiety [2]. Among other points of critique, the DSM-IV Hypochondriasis diagnosis has been criticized for being poorly and arbitrarily defined overlapping with other somatoform and psychiatric disorders [1], [4], [5], [19], [20]. In an earlier study based on the same patient sample as the present one, we showed that the recently defined diagnosis overcomes many of the drawbacks of the current DSM-IV diagnosis [2]. It is based on positive identification instead of exclusion of physical disease and is easy to use in clinical practice [2]. An important validation criterion for a diagnosis is its ability to predict outcome and to identify patients in need of treatment [40]. This study shows that the new, empirically established Health anxiety diagnosis identified a patient group with an unfavorable outcome. Furthermore, the severe Health anxiety patients used significantly more overall health care than patients with a well-defined medical condition, whereas this was not the case for DSM-IV Hypochondriasis patients. This finding is remarkable, as the DSM-IV criteria, contrary to the new criteria, require illness duration of least 6 months. Patients fulfilling the DSM-IV criteria thus ought to be more chronically ill. Furthermore, the DSM-IV criteria identified fewer patients than the new criteria for severe Health anxiety. This would usually indicate that the DSM-IV criteria identify more severely ill patients, but the results of this study showed that this was not the case. We did not find any differences in outcomes between patients with DSM-IV Hypochondriasis only, patients with severe Health anxiety only, and patients with both diagnoses. We did not test the difference in mean cost between Health anxiety and DSM-IV Hypochondriasis directly. These findings favor the validity of the new diagnostic construct. However, it has to be acknowledged that the validity of the new criteria has not yet been confirmed in independent populations.

We showed a fall in Health anxiety scores on the Whiteley-7 scale from index to later follow-up times for all patient groups. Whether they also had elevated scores prior to the consultation is unknown, but studies have indicated that high illness worry initiates help-seeking behavior [41], [42]. Thus, the patients may actually have been reassured by their doctor, but the Health anxiety patients were only partially reassured as they continued to have much higher Health anxiety scores after the consultation than patients with a well-defined medical condition. This is in line with the study of Lucock et al [43] who showed that the effect of reassurance after gastroscopy showing no serious illness was only temporary in patients with a pre-examination high level of Health anxiety.

The persistence of Health anxiety, which in this study was indicated by the high Health anxiety follow-up scores on the Whiteley-7 scale, seems to be in accordance with other studies reporting that many patients still fulfilled diagnostic criteria for Hypochondriasis during the follow-up period [15], [17]. To our knowledge, our study is the first to examine other longitudinal outcome measures than diagnoses.

### Impact of comorbidity on outcome

We have controlled for comorbid depression or anxiety disorder in the analyses. The poor outcomes for patients with Health anxiety or DSM-IV Hypochondriasis could not be explained by a comorbid mental disorder. The association between Health anxiety and unfavorable outcome may be explained by the possibility that patients with a well-defined medical condition are more inclined to fear for their health and therefore mistakenly may appear to have Health anxiety. However, we also adjusted for a comorbid medical condition according to the FPs, and we did not find that this had impact on outcome in the Health anxiety patients.

It is noteworthy that the severe Health anxiety patients had worse outcomes on any outcome measures used in this study, including PCS, than patients with a well-defined medical condition. The patients with severe Health anxiety scored from 6.0 to 7.2 lower on the PCS and from 8.8 to 12.6 lower on the MCS (unadjusted scores Table 4) compared with the medical condition patients and the general population sample. Such a difference is usually considered clinically significant, and for the MCS, the difference exceeded one SD. The MCS scores are below the scores for most disorders or diseases for which figures are available except for depression [27], and they are in particular much lower than reported in studies on Somatization disorder [44]. Health anxiety patients had worse or similar PCS scores than reported in studies on cancer, lower back pain, diabetes, and Somatization disorder patients but not quite as low as reported in rheumatoid arthritis and lung disease patients [27], [44]. We showed an improvement in PCS scores at later follow-up in Health anxiety as opposed to Dickinson et al [44] who showed a decrease in Somatization disorder patients.

We chose to include a control group consisting of patients, whom according to the FPs primarily consulted due to medically well-defined physical conditions. This choice offered the opportunity to compare the outcomes in Health anxiety patients with the outcomes in patients, who, for the major part, did not have a mental illness or a chronic medical condition and who did not consult due to medically unexplained symptoms. However, the control group was not representative of the primary care population as such as a large segment of patients likely to generate high health care costs and show great impairment was excluded. Thus, we only included about half of the patients approached for the study because of our selection of control group. The relevance of choosing this comparison group may be questioned as may the choice of any comparison group. The patients with a well-defined medical condition are a very heterogeneous group including patients with severe physical disease and patients consulting for minor or transient ailments, e.g. flu. Despite this, it is a more homogeneous group than the overall group of all patients consulting their FP, and we feel that using this group for longitudinal comparison makes sense and provides transparency. We also included an age and gender matched general population sample for comparison, but since we do not have follow-up data on this cohort, we could not use it for longitudinal comparison. Still, our choice of comparison group provides a notion of the primary care representatives and the Danish population compared with other countries. It would be interesting to compare the outcomes in patients with depression, anxiety, medically unexplained symptoms or other somatoform disorders and patients with chronic medical conditions with the outcomes of Health anxiety patients, but this calls for another study with focus on this comparison, and it is beyond the scope of the present paper.

### Strengths and limitations

A limitation of the study is the response rate at follow-up in some of the patient groups. The lowest response rates at two-year follow-up were in the DSM-IV Hypochondriasis group, in which 66% responded to the Whiteley-7 questionnaire and in the medical condition group in which 60% responded to the SF-36. The response rate was higher at all time points in all of the other patient groups. We did not detect any statistically significant differences between completers and non-completers of the follow-up questionnaire, and hence we do not expect any systematic bias as to non-completers. A strength of the study is that we had complete health care utilization data for all patients as the data were obtained from patient registers.

A major strength of this study is that the Health anxiety/Hypochondriasis diagnoses are based on the state of the art SCAN interview performed by psychiatrically trained physicians. However, we only interviewed a stratified sample of 701 patients of which 296 had either a Hypochondriasis/Health anxiety diagnosis or a well-defined medical condition according to their FPs. This means that some of the patients in the well-defined medical condition group were not interviewed, because they had low scores on the screening questionnaire and were not randomly selected for interview. Some of these non-interviewees may therefore have had undetected Health anxiety, and we estimate that approximately 29 patients did, which entails a misclassification bias. These 29 patients ought to have low scores on illness worry, and/or high self-rated health, and/or low use of health care for a differential misclassification bias to be present. We find it very unlikely that a patient with severe Health anxiety, and thus with symptoms that severely disturb or significantly interfere with everyday activities, has low illness worry, and/or high self-rated health, and/or low health care costs. We therefore assess that a misclassification bias would be of a non-differential type.

This study was a part of an RCT intervention study on medically unexplained symptoms and somatoform disorders in primary care [35], [36]. The intervention did not specifically address treatment of Health anxiety. Thus, we did not find any clinically or statistically significant treatment effects. Although insignificant, we cannot completely rule out that the outcomes may have been better as a result of the doctors' training and the intensive focus on the subject in the study period thus perhaps resulting in a bias towards an overly optimistic outcome. The FPs judged that about half of the Health anxiety patients attended either definitely or probably due to a medical condition. This indicates that the FPs need better procedures for identifying patients with Health anxiety, although we cannot conclude this for certain. Patients with Health anxiety may have consulted their FP due to a comorbid physical condition, and we did not register the primary reason for the consultation when a patient had more than one illness.

Another limitation of the study is that we did not re-examine the patients by means of the SCAN interview at follow-up as the follow-up was based solely on self-reported questionnaires (the SF-36 and the Whiteley-7) and health care costs from patient registers. Therefore, we do not know if any patients had developed another physical or mental disorder that may have influenced the outcome, for example whether part of the increase in health care costs may be attributable to an instable medical condition that surfaced after inclusion in the study. We also do not know if the patients still fulfilled the diagnostic criteria for Hypochondriasis after two years. A previous report has found that two thirds continued to meet DSM-IV criteria for Hypochondriasis after one year [17]. Another study found that 63% still met the criteria four to five years later [45]. We realize that continued fulfilment of the diagnostic criteria is an important outcome measure, and it is a limitation of this study that we only have self-reported Health anxiety at follow-up instead of new diagnostic interviews. However, we believe that prediction of outcome in the form of disability, subjective suffering, and health care costs may be more relevant outcome measures than patients fulfilling diagnostic criteria at follow-up. The mild Health anxiety diagnosis seems to have only a small impact on outcome. The mild Health anxiety patients' physical component scores were at most time points similar to those of the medical condition group, and their health care costs compared with the medical condition group were significantly lower both before and after index indicating a good outcome. Mild Health anxiety does thus not seem to be a precursor of severe Health anxiety. This is in accordance with the results of Barsky et al [16], who found that only one out of 24 patients with transient Hypochondriasis (i.e. Hypochondriasis with a duration of less than 6 months) developed Hypochondriasis according to DSM-III-R criteria at averagely 22-month follow-up. However, due to the small number of patients with mild Health anxiety, the outcome for this group should be taken with some reservation, in particular as to costs.

In the statistical analyses, we did not correct for multiple testing.

### Conclusions and implications

Health anxiety seems in its severe form to be a quite disturbing and persistent condition entailing high health care costs, and it should be more consistently diagnosed and aggressively treated. Studies now indicate that the condition can be treated effectively by specialists [46], [47]. However, as Health anxiety is very prevalent it is important that also FPs and other physicians achieve basic skills in treating and identifying the disorder. If patients' health worries are addressed at an early stage by the physicians, repeated consultations and medical examinations may be prevented, and it could also prevent development of more severe Health anxiety that needs intensive intervention.

This study adds further support to the validity of the recently introduced new criteria for severe Health anxiety, whereas the mild Health anxiety group may be less clinically relevant as these patients have a better outcome.

## Supporting Information

Table S1 Suggested diagnostic criteria for Hypochondriasis.
(0.03 MB DOC)
