# Supplementary material for: The Outcome of Health Anxiety in Primary Care. A Two-Year Follow-up Study on Health Care Costs and Self-Rated Health
Source: PLoS One. 2010 Mar 24;5(3):e9873. doi: 10.1371/journal.pone.0009873 (PMC2844425; doi:10.1371/journal.pone.0009873)
Supplement: Table S1 — Suggested diagnostic criteria for Hypochondriasis. (0.03 MB DOC) [file pone.0009873.s001.doc]

Table S1. Suggested diagnostic criteria for Hypochondriasis

| **A** |  | Obsessive *rumination* with intrusive thoughts, ideas or fears of harboring illness that cannot be stopped or can only be stopped with great difficulty |
| --- | --- | --- |
| **B** |  | | One (or more) of the following 5 symptoms | | --- | |
|  | 1 | **Either a or/and b.**  **a**. Worries about or preoccupation with fears of harboring a severe physical disease, or   thoughts about contracting a disease in the future, or preoccupation with other health  concerns.  **b**. Attention and increased awareness about bodily functions, physical sensations,  physiological reactions or minor bodily problems that are misinterpreted as serious disease. |
|  | 2 | Suggestibility and auto-suggestibility. If the patient hears or reads about illness (s)he is inclined to fear that (s)he has the same disease. |
|  | 3 | Excessive fascination with medical information |
|  | 4 | An unrealistic fear of being infected or contaminated by something touched, eaten or a person met. |
|  | 5 | Fear of taking prescribed medication |
| **C** |  | In case of the presence of a medical condition, the patient’s reaction clearly exceeds what would be expected from the medical condition alone. |
| **D** |  | The symptoms are not better explained by another mental disorder. |
| **E** |  | The symptoms should be present for most of the time for at least 2 weeks. |
| **F** |  | Specification:  Severe: At least one of the symptoms A-B is severely disturbing or significantly interfering with everyday activities.  Mild: All others. |
